# Check-In: An Educational Activity to Address Well-Being and Burnout among Pharmacy Students

**DOI:** 10.3390/pharmacy8040184

**Published:** 2020-10-08

**Authors:** Heidi V.J. Fernandes, Cynthia Richard, Kaitlin Bynkoski, Becky Ewan, Sherilyn K.D. Houle

**Affiliations:** School of Pharmacy, University of Waterloo, Waterloo, ON N2L3G1, Canada; heidi.fernandes@uwaterloo.ca (H.V.J.F.); c25richa@uwaterloo.ca (C.R.); kaitlin.bynkoski@uwaterloo.ca (K.B.); bewan@uwaterloo.ca (B.E.)

**Keywords:** burnout, pharmacy education, students, active learning, mental health, resilience

## Abstract

*Background:* Chronic workplace stress that has not been adequately managed can result in burnout. Healthcare providers; including pharmacists, may be particularly susceptible to this phenomenon, prompting the School of Pharmacy at the University of Waterloo to develop an active-learning activity to teach and reflect on healthcare provider burnout, called Check-In. *Methods:* Check-In was comprised of a 20 min online lecture on healthcare provider burnout, two pre-readings that highlighted burnout among physicians, and an optional one-on-one session between individual students and a faculty or staff member. A reflection guide was also shared among students and facilitators where students had to rate their current mental health on a 10-point scale and reflect on questions focusing on energy expenditure, self-care, and self-compassion within the past, present, and future. *Results:* Check-In was rewarding and overall positive for students and faculty. The personal connection with members from the school and the strategic timing of the activity within the curriculum notably contributed to the success of the activity. The short duration of individual sessions was the key criticism of the activity. Further research at the University of Waterloo School of Pharmacy will be explored to assess the long-term impact of Check-In on student well-being.

## 1. Introduction

Burnout is an occupational phenomenon recognized by the World Health Organization [1] and is included in the eleventh Revision of the International Classification of Diseases. Burnout is defined as a syndrome resulting from chronic workplace stress that has not been successfully managed [2], and is characterized by:

- “Feelings of energy depletion or exhaustion” [2];
- “Increased mental distance from one’s job, or feelings of negativism or cynicism related to one’s job” [2];
- “Reduced professional efficacy” [2].

While burnout can affect anyone, it is markedly found in professions that focus on caring for others, such as social workers, nurses, and doctors whose daily work involves demands and interactions with people having physical and emotional needs [3]. Burnout in physicians can be linked to patient dissatisfaction, medical error and malpractice risk, and higher turnover rates [4]. In 2018, the Canadian Medical Association’s National Physician Health Survey found that burnout and lower self-reported resilience were more frequent among residents than physicians, and among women compared to men [5]. Although much of the literature on healthcare provider burnout focuses on physicians, a recent study found that about half of health system pharmacists in the United States reported a high degree of burnout on at least one subscale of the Maslach Burnout Inventory Human Services Survey [6]. Pharmacy organizations have also increasingly prioritized the importance of well-being among pharmacists. For example, the American Association of Colleges of Pharmacy addressed the importance of investing in professional development that includes components of well-being and resilience during their 2019 annual meeting [7], and the Canadian Pharmacists Association hosted an online webinar for pharmacists on the topic of resilience in December 2019 [8].

Previous studies on pharmacy students’ mental health have attempted to capture student stress levels or well-being at a particular point in time, finding that pharmacy students experience academic distress and worse mental health, including maladaptive coping strategies, depression, and anxiety [9,10,11], which can vary depending on the student’s year in the program [11,12,13]. Mindfulness training has been applied to pharmacy students by two studies. The first, by Zollars et al. in the United States, found that use of the meditation app Headspace^TM^ (Santa Monica, CA, USA) enhanced mindfulness and students’ mental well-being, while also decreasing perceived stress [14]. The authors concluded that the use of this app can help students cope with the demanding nature of pharmacy undergraduate programs [14]. In Ireland, the curriculum-guided use of the mindfulness-based stress reduction (MBSR) program was found to significantly improve mental distress and stress of female pharmacy students [15]. While the Maslach Burnout Inventory was one of the measures employed by this study to assess the effectiveness of the program, the MBSR program does not specifically teach learners about burnout [16].

At our institution, the University of Waterloo, a President’s Advisory Committee on Student Mental Health investigated student mental health and recommended that wellness activities be integrated into the curriculum [17]. However, to date, students in our program received only minimal instruction related to wellness and coping strategies. Given its significance to practice and susceptibility among new practitioners, as well as our institution’s internal objectives, strategies to identify and address burnout may be a beneficial component of health professional education programs. To our knowledge, there are currently no studies focused on educating pharmacy students specifically on healthcare provider burnout. The purpose of this article is to share our experience of creating an active learning activity focusing on healthcare provider burnout, including its successes and limitations, in order to cultivate resilient future pharmacy professionals and inspire other pharmacy educators to adopt similar educational activities.

## 2. Materials and Methods

### 2.1. Development

The educational activity, referred to as Check-In, was developed originally by teaching and administrative staff at the University of Waterloo School of Pharmacy. It was inspired by individual check-ins performed by the Faculty of Medicine at the University of Alberta, of which information was obtained via personal communication. Additional resources used to cultivate the activity included the online guide “From Surviving to Thriving: Developing Personal and Academic Resilience” [18] designed to develop resilience among first-year university students, and Dr. Dike Drummond’s article “Physician Burnout: Its Origin, Symptoms, and Five Main Causes” [19]. Development of the activity was performed in collaboration with staff and faculty, which included the Associate Director of Curriculum, Director of Undergraduate Affairs, and Academic Advisor/Undergraduate Administrative Coordinator. As this activity was part of the third year Professional Practice lab-based course, we attempted to create student instructions and background preparation material consistent with those used for other lab activities. After these materials were created, a group of 20 fourth year PharmD students (e.g., the cohort senior to the students who were offered this activity) were invited to a focus group to gather their thoughts and feedback on the activity. Using their feedback in discussion with the aforementioned staff and faculty collaborators, the details of the educational activity were finalized.

### 2.2. Educational Activity

Within each academic term, students complete a course within the Professional Practice series that builds upon didactic therapeutic learning using applied, interactive laboratory activities. These lab activities simulate scenarios students may encounter in practice, refining students’ communication, drug information, and clinical decision-making skills. This series of courses is amenable to active-learning approaches and aims to equip students with practical skills as patient care providers. As such, an educational activity was implemented in the Winter 2020 offering of the Professional Practice 5 course to teach third-year pharmacy students about personal wellness and healthcare provider burnout. The overarching intention of the activity for the students can be summarized with the phrase: *you have to take care of yourself before you can take care of others*. The learning objectives for Check-In were to:

- Understand the relevance of healthcare provider burnout as a future pharmacy professional;
- Reflect on your current experience in the pharmacy program;
- Assess your current coping skills/strategies and recognize its sustainability and impact on your life after graduation.

Consistent with the activity offered by the University of Alberta, this Check-In included an in-person component. This format was chosen for a number of reasons; namely, an in-person activity provided a sense of accountability, potentially allowed for deeper self-reflection from the student, and allowed for the active provision of support or referrals to other resources as required. Additionally, our curriculum utilizes written reflections frequently (with two written reflections as part of this course alone), so we wished to avoid reflection fatigue [20].

All students were scheduled for a session on the day their professional practice lab would normally be held. Sessions were 15 min in length and consisted of a one-on-one interaction between a staff or faculty member from the School of Pharmacy and a student. Given the potentially sensitive nature of the activity, it was not mandatory, and students could opt out for any reason without any academic consequences, as no grades were associated with the activity. Students could also opt to switch facilitators from the one assigned and opt to do their Check-In along with a classmate, rather than alone, for a total of 30 min.

Prior to the activity, all students were encouraged to view a 20 min online lecture on healthcare provider burnout, and were provided with two pre-readings [19,21], whose author is from The Happy MD (www.thehappymd.com)—a website with resources on physician burnout. Viewing of these resources was also optional, but was highly encouraged, even if students opted out of the in-person component of the activity.

Although every student could choose to talk about any topic(s) of their choosing, a discussion guide was created to assist students and facilitators with initiating dialogue and guiding deeper self-reflection by the student. Students had access to this guide prior to the Check-In, allowing them to self-reflect before their session and think about topic areas they wished to structure the discussion around. Check-In was strategically scheduled to take place after a midterm exam in another course to capture students’ attention at a stressful time. Therefore, the guiding questions generally focus on their experiences in the past week.

At the start of Check-In, students were asked to rate themselves on a mental health numeric rating scale (Table 1), modified from an online scale [22] to improve its applicability to pharmacy students. This scale is not validated, and its primary purpose for Check-In was to quickly gauge the student’s well-being at the start of the activity. It also served a secondary purpose of allowing the facilitators to debrief after the activity and quantify how the student’s well-being was overall.

**Figure 1:**
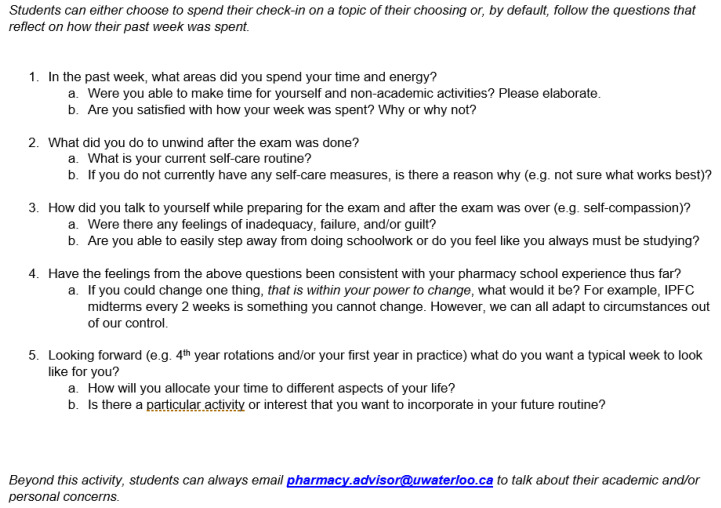
Part 2 of Check-In guide. Note: IPFC = integrated patient-focused care (a series of 9 extensive courses that integrate pathophysiology, pharmacotherapeutics, and pharmaceutical sciences).

Following completion of the mental health numeric rating scale, facilitators used a semi-structured series of questions to probe further and stimulate discussion. The first three questions were related to energy levels, self-care, and self-compassion, respectively, based on students’ experiences in the past week. The last two questions related these experiences to their past and looking forward to the future. Each main question included one or two follow-up questions, as indicated in Figure 1.

#### Required Resources for the Activity

With potentially 120 students to see on one day, we required the use of several faculty/staff members from the School of Pharmacy. In addition to the course coordinator, five other members were recruited for a total of six facilitators. The other five facilitators were chosen as individuals with an approachable and caring demeanor, who are also heavily involved with the well-being and advancement of our students, including:

- Associate Director of Curriculum;
- Teaching Fellow;
- Professional Practice Lab Coordinator;
- Director of Undergraduate Affairs;
- Academic Advisor/Undergraduate Administrative Coordinator.

Each facilitator was asked to be available for the whole day (8:30 a.m. to 3:30 p.m., divided into three two-hour lab sections with a 30 min break in between), and to provide use of their own private office in order to maintain privacy and confidentiality. All six facilitators met two weeks prior to go over logistics and expectations for Check-In.

In addition to our Check-In guides, on-campus and off-campus resources, such as workshops and crisis lines, were also made available to the students via pamphlets and materials that were at every session. Referrals to the university’s counselling services could be made by either the facilitator or student. Limited same-day appointments were available, any students found to be in active crisis would automatically be referred to see a counsellor.

### 2.3. Methods of Assessment

As this activity was deemed optional, there was no summative assessment of student learning. A follow-up survey link was posted to all students using the university’s online learning platform and was open to complete from March 12th to April 30th, 2020. The aim of the survey was to gain a better understanding of, and feedback on, the activity from the students’ perspective. This survey received ethics approval from the University of Waterloo Research Ethics Committee (ORE #41852) and was administered online via Qualtrics^TM^ software (Qualtrics, Provo, UT, USA). Questions consisted of open-ended response questions, Likert scales, and rankings. Completion of the survey was voluntary, and students were allowed to skip any questions that they wished. As an optional and ungraded activity, there were no academic consequences of completion or non-completion. The survey was designed to be inclusive of both students that did attend Check-In and did not attend to ascertain all students’ views on wellness activities. However, demographic information was only obtained for those that participated in the activity. Descriptive statistics were performed for the quantitative questions using Microsoft Excel for Windows 10, version 1902 (Redmond, WA, USA) and the qualitative free-text responses were collated and assessed for general feedback about the activity. All analyses were performed by the course coordinator and were performed following the completion of the course.

## 3. Results

Nearly two-thirds of the cohort (76 students out of a possible 120, 63%) participated in Check-In. The survey received 63 responses, with 24 discarded as they were incomplete. Of the remaining 39 responses, two participants did not consent to participate. The analysis of results included 37 responses, out of a possible 120, which were completed and had consent provided.

### 3.1. Students Who Did Attend Check-In

Of the completed responses, 33 respondents did attend Check-In (43% of attending students) and their demographics are summarized in Table 2.

Check-In was well-received by students. More than half of the respondents who attended the activity agreed that it positively contributed to their wellness as a student and future pharmacist, with increased self-awareness on burnout and its relevance for healthcare professionals. Although students’ opinion on whether Check-In should be mandatory was mixed, 82% either agreed or strongly agreed that similar wellness activities should be embedded in the curriculum. A few survey responses (*n* = 4) mentioned that Check-In should have been done earlier in their program or throughout their time as a pharmacy student, rather than just in their third year. However, appreciation of this activity was still widely cited in the survey, with some selected free-text responses listed below:

- “It very much seemed like an activity meant to help us, which was a relief. It underscores that the School of Pharmacy considers its students [as] more than just numbers”.
- “I appreciate the addition of the mental health check in for the program. I think it’s so important to check in with the students face to face and see how they are doing”.

The biggest limitation for Check-In was time allotment, as 15 min proved to be too short for both students and the facilitators. It was most commonly cited for what students did not like about the activity. As one student put it, “With such little time there was a lot that was kind of left unsettled and was unsettling for the rest of the day.”

Table 3 summarizes the overall impact of Check-In. The performance of the facilitators was a strength of this activity as 78% of students either disagreed or strongly disagreed that faculty or staff should *not* be used for this activity, and 97% of respondents felt comfortable with their facilitator and indicated that the faculty or staff member adequately facilitated their Check-In. When asked what they most liked about the activity, respondents most often cited that they liked the ability to talk personally to a faculty/staff member and express their concerns. As one student mentioned, “I liked the one on one environment that allowed me to speak freely about my thoughts on burn-out and general experiences I had throughout the course of pharmacy school.” Having the faculty and staff members be at the forefront of the activity also demonstrated to the students how much the school cares about their well-being. Some comments alluding to this are listed below:

- “I love [Check-In] existed in the first place. I love that the school is taking the initiative to check in on us, and that you made every effort to make us comfortable”.
- “I know many positions at the school of pharmacy are time consuming and demanding but the fact they took time to talk to students made it feel like I was taken seriously”.
- “The faculty member is not completely removed from the pharmacy environment so they are able to provide valuable insight and are able to still relate to my experiences as a pharmacy student compared to someone that is not a part of the pharmacy world, that is not part of Waterloo”.
- “I felt genuinely cared for by the prof assigned to me, and in turn the school of pharmacy for letting something like this be a part of the course”

Overall, each aspect of Check-In, including background reading materials, question guide, facilitators, and organization of the activity, was well-received, with over half of students rating each of these as either very or extremely effective (see Table 4). The in-person components of the activity were rated more highly than the background reading and the question guide, highlighting the importance of the one-on-one interaction. Similar thoughts about the question guide, particularly with the mental health numeric rating scale, were also raised during the debrief among facilitators. Doubts were raised about the usefulness of the scale for the overall objective of Check-In, and its placement right before questions was found to be awkward. While the scale and guided questions were a helpful starting point and could frame the rest of the conversation, facilitators noted greater connection and more meaningful conversations when they deviated from the guide and asked their own questions.

When asked to rate their self-perceived knowledge and skills regarding Check-In’s learning objectives on a sliding scale from 0: poor to 100: excellent, students averaged 79.8 (SD = 10.2) for their understanding of healthcare provider burnout and 78.6 (SD = 17.3) on their ability to self-reflect on current wellness practice(s) and well-being following the activity. The free-text responses demonstrated that students gleaned the relevance of healthcare provider burnout through the Check-In activity and the importance of wellbeing for their future practice, with some selected responses listed below:

- “It’s important to take care of yourself and it’s more than okay to put your well-being first”.
- “Burn-out is so much more common than you’d think, even if you’re not experiencing it right this second or even expecting it in the future”.
- “I realized it was a very important moment of pause that I could take to really reflect on my own mental health amidst the [busyness] that is pharmacy school. It gave me a chance to get my bearings, make myself aware of my own thoughts and environment and to re-ground myself”.
- “We cannot pour from an empty vessel and so it’s fundamental that I invest more into myself so I can properly help serve others well”.
- “It was really helpful in making me more aware of my mental health and about [healthcare professional] burn-out in general. It is good to be able to talk seriously about mental health without stigma”.
- “It is important to recognize that healthcare provider burnout affects everyone at some point in their career. It is important to separate work life from home life, and develop regular self-care habits to reduce healthcare provider burnout”.

### 3.2. Students Who Did Not Attend Check-In

Of the students who completed the survey, four respondents did not attend Check-In (9% of non-attending students). Despite their absence, all students either agreed or strongly agreed that wellness activities should be incorporated into the curriculum. Students most commonly cited that they decided to not attend Check-In to instead use the time to do something else (e.g., catch up on schoolwork). Two students also said they have strong support systems or outlets to release stress personally but find that wellness activities would still benefit many other students. As one student wrote, “Personally, I have other outlets in my life that I use to release tension or frustration, however I think that wellness activities are helpful for many other students and so they should be incorporated as optional activities into the curriculum.” All students still appreciated having this activity available to them, as captured by one student “I’ve found that in this program it is really hard to slow down enough to really register how my mental health state is. It was really refreshing coming into this semester and being told ‘Hey this day of the semester had been set aside for you to check in with yourself and/or someone on staff and see how you are doing’”.

## 4. Discussion

This educational activity was rewarding for both students and facilitators. To our knowledge, this is the first published active-learning educational activity focusing on healthcare provider burnout in a pharmacy program. While check-ins may have been performed in health professional schools before, no publications describing their structure or impact were identified. Furthermore, previous studies of student wellness focused on determining general student well-being at a point in time rather than the development of an active-learning strategy to address it and provide education on the prevention and detection of burnout.

A number of factors are believed to have contributed to the success of this activity. First, leadership at the School of Pharmacy identified student wellness as an area of improvement and supported the development of this educational activity, including the utilization of faculty and staff as facilitators. Involving leadership and targeting burnout during professional school have been identified as strategies to combat burnout [24]. Indeed, a recent study identified that faculty help and institutional resources were a key strategy in improving pharmacy students’ wellbeing [25]. Furthermore, student engagement was encouraged by having active faculty involvement, integrating the activity within a course during class time, and the intentional scheduling of the activity following a potentially stressful exam when students may be more receptive to the importance of maintaining personal wellness. These strategies may have helped our program overcome some of the limitations observed by O’Driscoll et al., and their paper describing an online mindfulness intervention offered at a pharmacy school in Ireland [26], where the intervention was delivered without an in-person component and offered by a researcher unfamiliar to the students and from a different institution, whose face-to-face interaction was limited to recruitment at best. Indeed, students in that program mentioned the importance of a “whole-school” approach to implement wellness training activities [26]. In a demanding program, having this week of the course dedicated to self-care rather than offering the activity as an optional addition to existing workload allowed students to more easily integrate it into their schedule. Efforts were also made to limit the time investment required to participate to approximately 1.5 h total, in comparison to the four-week curriculum of the mindfulness-based stress reduction (MBSR) program [26].

Check-In’s feedback survey was administered while our institution transitioned all courses online due to COVID-19 and the results obtained may have been affected as a result. A limitation of the feedback obtained from our survey was self-selection bias, which has been reported in previous studies [15,27]. With a response rate of 28% (33 students who did Check-In and responded to the survey, out of a possible 120), we cannot assume these findings are generalizable to the entire cohort of students. We also did not have a sufficient number of responses from students who did not attend the in-person component of the activity to compare results between attendees and non-attendees. Without other published literature on similar programs and with limited resources to refer to during its creation, there are improvements that can be made in further offerings. Primarily, the length of time for the student’s one-on-one sessions needs to be lengthened as 15 min proved to be too short for both students and facilitators. Each cohort has 120 students and, although attendance would not be mandatory, the activity needs to be planned as if all students would attend, requiring twice the number of facilitators. Doubling the facilitators from six to twelve would allow not only for better time management but would also provide the students a greater number of facilitators to choose from; however, recruitment of this many individuals with interest and availability would be a challenge. While it was a rewarding experience to dedicate their day to students, it is a big commitment and not all faculty may share the same sentiment. Voluntary recruitment of faculty facilitators and clear expectations regarding the nature of the activity may help faculty understand if partaking in this activity is of interest to them. As students commented positively on the involvement of faculty and staff as facilitators, and found the interactions to be friendly and supportive, future offerings will continue to apply the same criteria for facilitator selection: a teaching/support role at the school, and a caring and approachable demeanor. The addition of a more specific and validated tool than the mental health numeric rating scale, such as the Maslach Burnout Inventory [28], may offer greater insight into a student’s current mindset. Students could complete the inventory prior to Check-In, in addition to viewing the other background materials, to get a truer sense of how they are feeling.

Student feedback indicated that overall, Check-In was an effective teaching strategy for addressing student and healthcare professional burnout and well-being and provided valuable takeaways. Feedback suggests that it would be beneficial for check-ins to be initiated earlier in the program and maintained throughout.

## 5. Conclusions

Sharing our activity and its evaluation within the school, the university, and the academic community as a whole, we hope that this activity encourages other professors from all disciplines to think about students’ wellness and how it can be addressed within their courses. Future research will seek to interview participating students at two points in time to assess the longer-term impact of the activity.

## Figures and Tables

**Table 1:** Mental health numeric rating scale for Check-In activity.

Rating	Description
1	This past week was a-okay! There is absolutely nothing wrong with how the past week was spent.
2	You’re a bit frustrated or disappointed with this past week. However, you’re able to bounce back from the negative really quickly.
3	Things have bothered you in this past week, but you’re coping. You might be overtired or your diet may have changed while studying for the midterm (e.g., emotional equivalent of a headache).
4	This week was a bad week. You still have the skills to get through it, but be kind to yourself.
5	Your mental health and/or the pharmacy curriculum are starting to impact on your everyday life. Easy things are becoming difficult. It may be worth booking an appointment with counselling services and/or your doctor.
6	You can’t do your usual routine due to your mental health and/or pharmacy curriculum.
7	You’re avoiding things that make you more distressed, but that will make it worse. It has been affecting your school performance and/or your daily activities.
8	You can’t hide your struggles anymore. You may have issues sleeping, eating, having fun, socializing, and working/studying. Your mental health is affecting almost all parts of your life.
9	You’re at a critical point. You aren’t functioning anymore. You need urgent help and may be a risk to yourself and/or others if left untreated.
10	The worst mental and emotional distress possible. You can’t imagine things ever getting better and you are losing hope. Contact a crisis line immediately.

**Table 2:** Demographics of survey respondents attending Check-In (*n* = 33).

Characteristic	Frequency
**Age**	
22	7 (21%)
23	5 (15%)
24	8 (24%)
25	3 (9%)
26	4 (12%)
28	1 (3%)
29	1 (3%)
30	1 (3%)
Older than 30	1 (3%)
Did not answer	2 (6%)
**Prior Education**	
2 years of undergraduate education (Conditional Admission to Pharmacy student) [23]	3 (9%)
2 years of undergraduate education (non-Conditional Admission to Pharmacy student)	9 (27%)
3 or 4 years of undergraduate education (did not confer degree)	4 (12%)
Undergraduate degree completed	16 (48%)
Graduate degree completed	1 (3%)
**Gender**	
Female	24 (73%)
Male	9 (27%)
**Mental Health Numeric Scale Rating**	
2	8 (24%)
3	11 (33%)
4	8 (24%)
7	1 (3%)
8	2 (6%)
9	1 (3%)
10	1 (3%)
Did not answer	1 (3%)

**Table 3:** Student responses on overall impact of Check-In.

Check-In Positively Contributed to My Wellness as A Student				
Strongly Agree	Agree	Neither agree nor disagree	Disagree	Strongly Disagree
51.5% (*n* = 17)	27.3% (*n* = 9)	18.2% (*n* = 6)	0	3.0% (*n* = 1)
**Check-In will contribute to my well-being as a future pharmacist**				
Strongly Agree	Agree	Neither agree nor disagree	Disagree	Strongly Disagree
21.2% (*n* = 7)	48.5% (*n* = 16)	21.2% (*n* = 7)	3.0% (*n* = 1)	6.1% (*n* = 2)
**Check-In increased my self-awareness on burnout and its relevance for healthcare professionals**				
Strongly Agree	Agree	Neither agree nor disagree	Disagree	Strongly Disagree
39.4% (*n* = 13)	42.4% (*n* = 14)	9.1% (*n* = 3)	6.1% (*n* = 2)	3.0% (*n* = 1)
**Check-In should be mandatory for students**				
Strongly Agree	Agree	Neither agree nor disagree	Disagree	Strongly Disagree
27.3% (*n* = 9)	15.1% (*n* = 5)	18.2% (*n* = 6)	30.3% (*n* = 10)	9.1% (*n* = 3)
**UW School of Pharmacy should have similar wellness activities embedded into the curriculum**				
Strongly Agree	Agree	Neither agree nor disagree	Disagree	Strongly Disagree
60.6% (*n* = 20)	21.2% (*n* = 7)	12.1% (*n* = 4)	3.0% (*n* = 1)	3.0% (*n* = 1)
**The faculty members and/or staff for the Check-In activity adequately facilitated my Check-In session**				
Strongly Agree	Agree	Neither agree nor disagree	Disagree	Strongly Disagree
69.7% (*n* = 23)	27.3% (*n* = 9)	3.0% (*n* = 1)		
**Faculty members and/or staff should not be used as future facilitators for this activity**				
Strongly Agree	Agree	Neither agree nor disagree	Disagree	Strongly Disagree
3.0% (*n* = 1)	6.1% (*n* = 2)	12.1% (*n* = 4)	36.4% (*n* = 12)	42.4% (*n* = 14)

**Table 4:** Student responses on the effectiveness of each component of the activity.

Background Materials Posted on LEARN				
Extremely effective	Very effective	Moderately effective	Slightly effective	Not effective at all
18.2% (*n* = 6)	39.4% (*n* = 13)	42.4% (*n* = 14)		
Questions asked to self-reflect *				
Extremely effective	Very effective	Moderately effective	Slightly effective	Not effective at all
18.2% (*n* = 6)	36.4% (*n* = 12)	30.3% (*n* = 10)	9.1% (*n* = 3)	
**Facilitator**				
Extremely effective	Very effective	Moderately effective	Slightly effective	Not effective at all
42.4% (*n* = 14)	39.4% (*n* = 13)	12.1% (*n* = 4)	3.0% (*n* = 1)	3.0% (*n* = 1)
**Organization of the activity**				
Extremely effective	Very effective	Moderately effective	Slightly effective	Not effective at all
45.4% (*n* = 15)	39.4% (*n* = 13)	9.1% (*n* = 3)	6.1% (*n* = 2)	

* one missing response.
